# The Value of Punching It Out: Patient With Cowden Syndrome and MALT Lymphoma of the Lung

**DOI:** 10.1155/crom/3254725

**Published:** 2025-10-23

**Authors:** Allison Jay, Morgan Devlin, M. Susan Jay, Ava Powell

**Affiliations:** ^1^Department of Genetics, Henry Ford St. John, Detroit, Michigan, USA; ^2^Department of Pediatrics and Adolescent Medicine, Medical College of Wisconsin, Milwaukee, Wisconsin, USA; ^3^Department of Radiology, Henry Ford St. John, Detroit, Michigan, USA

## Abstract

**Introduction:**

Cowden syndrome (CS) is a phenotypic representation of PTEN hamartoma tumor syndrome. CS is the result of dysregulation of the MTOR pathway contributing to cellular proliferation, which leads to an increased risk for the development of benign and malignant tumors of the breast, thyroid, endometrium, and kidney. There are scarce reports of patients with this condition developing lymphomas.

**Case Presentation:**

We present an African American female with a history of MALT lymphoma of the right lung diagnosed at age 41 treated with Rituxan who then presented at age 48 with a triple-negative cancer of the right breast. Physical exam showed macrocephaly, thyromegaly, and palmar pits. Family history was notable for a sister deceased from ovarian cancer at age 21 and a mother deceased from colon cancer at age 61. Genetic testing via peripheral blood identified a heterozygous PTEN pathogenic variant p.R130Q consistent with a molecular diagnosis of CS. Skin biopsy was coordinated given concern the MALT lymphoma could have contributed to spurious results and confirmed the same pathogenic PTEN mutation.

**Conclusion:**

Lymphomas have rarely been reported with this condition although activation of the PTEN pathway has been previously reported as a contributing factor in B cell lymphoma. Skin biopsies may offer the best specimen for patients with hematologic malignancy.

## 1. Introduction

Cowden syndrome (CS) was first reported in 1963 and named after the family it was first reported in [1]. While BRCA1 and BRCA2, which occur in approximately 1/400 individuals, are well-known genes contributing to a high risk of breast cancer, CS which occurs in 1/200,000 is less well known [1]. CS and related syndromes characterized by germline mutations in the PTEN tumor suppressor gene are collectively known as PTEN hamartoma tumor syndromes (PHTS). A diagnosis of CS can be made based on clinical features or the identification of a pathogenic variant within the PTEN gene. Individuals with CS have up to an 85% lifetime risk of breast cancer, with approximately half of individuals presenting by age 50 [2]. Benign multinodular goiter has been reported in 75% of patients, with the risk of cancer of the thyroid ~35%. Ninety percent of patients with CS will have polyps, with ~9% developing colon cancer. The lifetime risks for kidney and endometrial cancer are 35% and 28%, respectively. Additionally, risks for secondary malignant neoplasms are increased over the general population. Ngeow et al. did a 7-year prospective study from 2005 to 2012 of 2192 adult patients with Cowden and found the cumulative 10-year risk of a second breast cancer was 29% [3].

Mucosa-associated lymphoid tissue (MALT) lymphoma, a subtype of extranodal marginal zone lymphoma (ENMZL), typically arises in the gastrointestinal tract but is also found in other mucosal sites such as the salivary glands and lungs. The pathogenesis of MALT lymphoma is often associated with chronic immune stimulation (such as bacterial infections in the stomach or lungs) or autoimmune disease [4].

Although PTEN dysfunction has been implicated in various hematologic malignancies, an association between CS and MALT lymphoma is rare, with few cases of lymphoma and Cowden documented in the literature [5, 6].

The standard approach for hereditary cancer testing includes multigene panel testing using next-generation sequencing. Multiple sample types are accepted for germline genetic testing and should be considered based on various factors. Most often, a purple top EDTA (whole blood) is obtained, and DNA is then extracted from the lymphocytes in the sample. However, considerations for the best sample type to use for germline genetic testing in a patient with a previous or active hematologic malignancy diagnosis include the pathophysiology, disease state, course of treatment, and remission status [7].

This case report describes a patient with molecularly confirmed CS who developed MALT lymphoma. We discuss considerations for genetic testing in patients with lymphoma and highlight the phenotypic features of CS as well as recommended cancer surveillance when caring for these patients and their families.

## 2. Case Presentation

**Figure 1:**
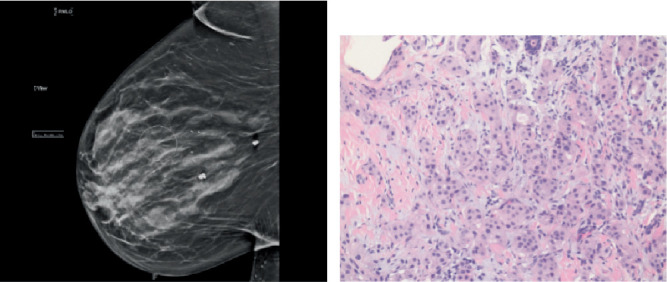
(a) Mammogram of the right breast showing an irregular hypoechoic mass at 10:00, 8 cm from the nipple measuring 1.7 × 1.2 × 1.7 cm. Architectural distortion is highlighted by the circular ring. (b) Pathology shows invasive ductal carcinoma.

**Figure 2:**
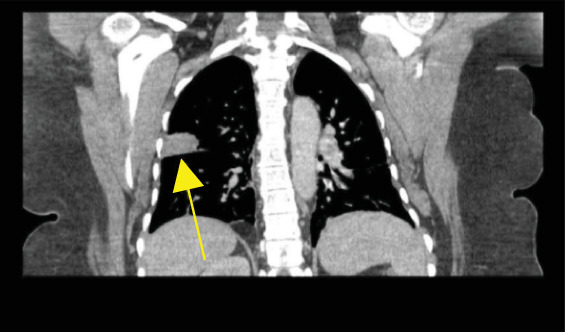
CT of the chest showing subpleural pulmonary opacity in the upper right lobe measuring 4.0 × 3.4 cm. Yellow arrow highlighting the right lung mass, biopsy proven MALT lymphoma.

A 48-year-old African American female underwent a screening mammogram that showed architectural distortion of the right breast. This was followed by a diagnostic mammogram, which revealed a lesion in the right breast 8 cm from the nipple measuring 1.7 × 1.2 × 1.1 cm (Figure 1a). Additional masses were also seen in the right breast. The 8-cm lesion was biopsied; pathology showed invasive ductal cancer with triple-negative hormone receptors (Figure 1b). Pertinent other oncological history includes a diagnosis of MALT lymphoma of the lung at age 41: lesion identified by CT (Figure 2) treated with Rituxan. She was not found to be a radiation oncology candidate; thus, radiation therapy was not completed. She had been in remission since that time.

Her past surgical history includes tubal ligation at 38, gastric bypass at 47, multiloculated benign phyllodes tumor, and anal lesion removed at age 46 consistent with HPV. Past colonoscopies showed multiple hyperplastic polyps of the colon and gastric tract. Prior CT identified enlarged thyroid with several calcified nodules present.

**Figure 3:**
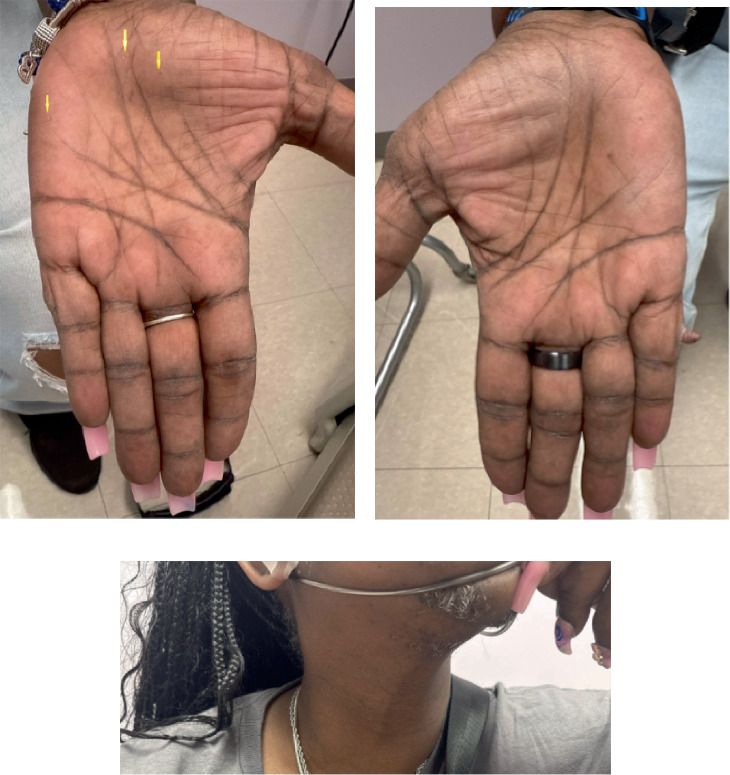
Clinical features of the patient include breast cancer, macrocephaly, and mucocutaneous lesions. (a) Palmar keratotic pits. (b) Keratotic horns of the right fifth digit. (c) Thyromegaly and small skin-colored papules (suspected trichilemmomas, not confirmed) of the neck.

Physical exam was notable for macrocephaly with a head circumference measured at 63.5 cm, thyromegaly, and palmar pits. Together, these findings confirmed a clinical diagnosis of CS (Figure 3).

Given her age at breast cancer diagnosis and triple-negative receptor status, this patient met the guidelines for hereditary cancer testing criteria by the National Cancer Comprehensive Network and was offered a multigene panel [8]. This analysis interrogated genes for high-penetrance breast cancer susceptibility including BRCA1, BRCA2, CDH1, PALB2, PTEN, STK11, and TP53. A blood sample was obtained and analyzed using next-generation sequencing for 77 genes associated with hereditary cancer. Results identified a heterozygous pathogenic variant in PTEN at a variant allele fraction of 43%. Due to her history of MALT lymphoma, fibroblasts were obtained via skin punch biopsy which confirmed germline origin of the PTEN pathogenic variant which led to an arginine at codon 130 being replaced by glutamine.

**Figure 4:**
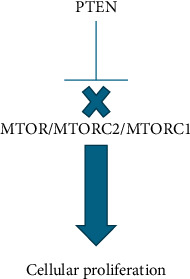
PTEN mutations contribute to cellular proliferation.

While the amino acids have similar properties, functional studies showed diminished phosphatase activity compared to patients with wild-type PTEN. The wild-type PTEN protein is a phosphorylase which dephosphorylates phosphatidylinositol 3,4,5-triphosphate (PIP3) and consequently inhibits the PI3K/AKT/mTOR signaling pathway. PTEN's lipid phosphatase activity is essential for its function as a tumor suppressor. The patient's missense mutation disrupts the phosphatase activity, therein diminishing PTEN's role as a tumor suppressor. [9].  Figure 4 demonstrates how loss of PTEN wild-type activity can lead to downstream cellular proliferation and contribute to cancer. Further, functional studies were done which found the mutation also impacted the cellular localization of PTEN compared to wild type [10]. Because the laboratory also noted that this pathogenic variant had been identified as a de novo mutation in a female with macrocephaly and breast cancer at age 29, this genetic finding was consistent with the patients clinical findings [11].

The patient is currently receiving chemotherapy for the treatment of her breast cancer, followed by consultation and evaluation for bilateral mastectomy. In concordance with NCCN management guidelines for individuals with CS, a gynecologic oncology referral was placed for endometrial cancer surveillance [8]. She was also counseled regarding risks and management for thyroid, renal, and colon cancer. Implications for family members were also reviewed.

## 3. Discussion

This case illustrates a rare presentation of MALT lymphoma in a 48-year-old patient with CS and judicious choosing of sample type for germline genetic testing in patients with a hematologic malignancy. Our patient had been in clinical remission for 6 years, so a blood sample was originally deemed suitable. A follow-up skin biopsy also confirmed the clinical exam findings and blood sample genetic result.

A review of the literature yielded scarce reports of lymphoma in Cowden patients. Elston et al. first reported non-Hodgkin's lymphoma in a 70-year-old patient with CS [6]. Hagelstrom et al. reported a 7-year-old patient with B cell lymphoma and then male breast cancer at age 31 [12]. One other case has been reported in the literature of a patient with CS and MALT lymphoma of the esophagus in a 60-year-old Japanese woman [13].

PTEN works as a tumor suppressor gene encoding the phosphatase and tensin homolog protein. This protein works to inactivate the PI3K/AKT cascade in the mTOR pathway to cause cell cycle arrest and apoptosis [14, 15]. Germline pathogenic variants in PTEN result in either truncated, dysfunctional, or a lack of PTEN protein leading to dysregulation of the mTOR pathway, and cellular proliferation can proceed unchecked (Figure 4) [16].

In somatic cancers, the function of the PTEN protein may be compromised by a variety of mechanisms such as epigenetic factors and tumor stage in addition to pathogenic variants [16]. Hollander observed that PTEN pathogenic variants have been identified at a lower frequency in lymphoid malignancies versus solid tumors [17]. The highest rates of PTEN pathogenic variants are found in T cell acute lymphoblastic leukemia, with fewer pathogenic variants in acute T cell lymphomas and large B cell lymphomas. Across the small B cell lymphomas, loss of PTEN activity is seen among follicular lymphoma (21% of cases) and mantle cell lymphoma (15% of cases) [18]. Vela et al. found that the most frequent genetic alterations were identified in the KMT2D, TNFAIP3, PRDM1, NOTCH1, and EP300 genes in pulmonary marginal zone lymphoma (MALT lymphoma of the lung). In their article, they outline that three patients with pulmonary MALT out of 28 patients had PTEN alterations, which is 11% [4].

In patients with a history of hematologic malignancy, genetic variants identified on germline testing may be somatic in origin; hence, there may be clinical cases where a skin punch biopsy may be preferable (Table 1). Hematologic malignancy–acquired events in the leukocyte lineages used for germline genetic analysis can make interpretation of identified variants complicated, as peripheral blood can contain tumor—or clonal hematopoiesis related acquired genetic variants [19] Thus, an alternative sample is recommended, such as skin fibroblasts, for confirmatory testing [7, 20].

Sarah Bannon, a certified genetic counselor who was at MD Anderson and a contributor to the Practice Resource of the National Society of Genetic Counselors, gave a lecture on September 10, 2020, for *I Care Genetics* on Inherited Blood Cancers. Bannon et al. coined the phrase “when in doubt punch it out,” which may be a memorable axiom for hematologists and oncologists when considering germline genetic testing.

Keeping this axiom in mind, despite the patient meeting defined criteria for a clinical diagnosis of CS, a skin punch biopsy was obtained to ensure the accuracy of molecular genetics analysis for appropriate counseling regarding recurrence risk and future cancer management and surveillance. The clinical team wanted confirmation given a bilateral mastectomy would be recommended for her breast cancer because of the high risk of a second breast cancer she would face if positive for Cowden. In conclusion, this patient case highlights the many cancers patients with Cowden may be at risk for and require surveillance and the importance of considering a skin biopsy for hereditary testing in those patients with underlying hematologic malignancy.

## 4. Conclusion

For patients with hematologic malignancy, skin biopsy may be the optimal source for genetic germline hereditary testing.

## Figures and Tables

**Table 1:** Hematologic conditions where skin biopsy may be best for germline testing.

**Patient history**	**Reason**
Allogenic bone marrow transplant from donor	Blood will test the donor's DNA
CLL	DNA is isolated from white blood cells, somatic mutations in lymphoid progenitor cells may lead to spurious results
AML	Somatic mutations in myeloid progenitor cells may make germline interpretation difficult
CML	Somatic mutations in myeloid progenitor cells may make germline interpretation difficult
NHL and HL Stage IV	Somatic mutations in B cell lymphocytes may contribute to spurious results not reflective of germline status
Clonal hematopoiesis, variant allele fraction ~20%–35%	Somatic mutations in circulating lymphocytes do not represent germline status

## Data Availability

Data are available on request due to privacy/ethical restrictions.

## References

[1] Dragoo D. D., Taher A., Wong V. K. (2021). PTEN Hamartoma Tumor Syndrome/Cowden Syndrome: Genomics, Oncogenesis, and Imaging Review for Associated Lesions and Malignancy. *Cancers*.

[2] Yehia L., Eng C., Adam M. P., Feldman J., Mirzaa G. M., Pagon R. A., Wallace S. E., Amemiya A. (1993). PTEN Hamartoma Tumor Syndrome. *GeneReviews®*.

[3] Ngeow J., Stanuch K., Mester J. L., Barnholtz-Sloan J. S., Eng C. (2014). Second Malignant Neoplasms in Patients With Cowden Syndrome With Underlying Germline PTEN Mutations. *Journal of Clinical Oncology*.

[4] Vela V., Juskevicius D., Dirnhofer S., Menter T., Tzankov A. (2022). Mutational Landscape of Marginal Zone B-Cell Lymphomas of Various Origin: Organotypic Alterations and Diagnostic Potential for Assignment of Organ Origin. *Virchows Archiv*.

[5] Galli E., Malafronte R., Brugnoletti F., Zollino M., Hohaus S., D'Alo F. (2020). Burkitt Lymphoma as Fourth Neoplasia in a Patient Affected by Cowden Syndrome With a Novel PTEN Germline Pathogenic Variant. *Mediterranean Journal of Hematology and Infectious Diseases*.

[6] Elston D. M., James W. D., Rodman O. G., Graham G. F. (1986). Multiple Hamartoma Syndrome (Cowden’s Disease) Associated With Non-Hodgkin’s Lymphoma. *Archives of Dermatology.*.

[7] Stewart B. L., Helber H., Bannon S. A. (2025). Risk Assessment and Genetic Counseling for Hematologic Malignancies-Practice Resource of the National Society of Genetic Counselors. *Journal of Genetic Counseling*.

[8] NCCN Clinical Practice Guidelines in Oncology Genetic/Familial High Risk Assessment: Breast, Ovarian, Pancreatic and Prostate.

[9] Han S. Y., Kato H., Kato S. (2000). Functional Evaluation of PTEN Missense Mutations Using In Vitro Phosphoinositide Phosphatase Assay. *Cancer Research*.

[10] Lobo G. P., Waite K. A., Planchon S. M., Romigh T., Nassif N. T., Eng C. (2009). Germline and Somatic Cancer-Associated Mutations in the ATP-Binding Motifs of PTEN Influence Its Subcellular Localization and Tumor Suppressive Function. *Human Molecular Genetics*.

[11] Mester J., Eng C. (2012). Estimate of De Novo Mutation Frequency in Probands With PTEN Hamartoma Tumor Syndrome. *Genetics in Medicine*.

[12] Hagelstrom R. T., Ford J., Reiser G. M. (2016). Breast Cancer and Non-Hodgkin Lymphoma in a Young Male With Cowden Syndrome. *Pediatric Blood & Cancer*.

[13] Mizuno S., Takeichi T., Sato J. (2018). Multiple Keratotic Papules and Plaques on the Trunk in Cowden’s Disease With MALT Lymphoma. *Journal of Dermatology*.

[14] Matsumoto C. S., Almeida L. O., Guimarães D. M. (2016). PI3K-PTEN Dysregulation Leads to mTOR-Driven Upregulation of the Core Clock Gene BMAL1 in Normal and Malignant Epithelial Cells. *Oncotarget*.

[15] Wang X., Huang H., Young K. H. (2015). The PTEN Tumor Suppressor Gene and Its Role in Lymphoma Pathogenesis. *Aging (Albany NY)*.

[16] Luongo F., Colonna F., Calapa F., Vitale S., Fiori M. E., De Maria R. (2019). PTEN Tumor-Suppressor: The Dam of Stemness in Cancer. *Cancers*.

[17] Hollander M. C., Blumenthal G. M., Dennis P. A. (2011). PTEN Loss in the Continuum of Common Cancers, Rare Syndromes and Mouse Models. *Nature Reviews. Cancer*.

[18] Karatrasoglou E. A., Dimou M., Piperidou A., Lakiotaki E., Korkolopoulou P., Vassilakopoulos T. P. (2023). The Role of mTOR in B Cell Lymphoid Malignancies: Biologic and Therapeutic Aspects. *International Journal of Molecular Sciences*.

[19] Jaiswal S., Ebert B. L. (2019). Clonal Hematopoiesis in Human Aging and Disease. *Science*.

[20] DeRoin L., Cavalcante de Andrade Silva M., Petras K. (2022). Feasibility and Limitations of Cultured Skin Fibroblasts for Germline Genetic Testing in Hematologic Disorders. *Human Mutation*.

